# Capacity of enforcers and level of enforcement of the Tobacco Control Act 2015 in Kampala, Uganda

**DOI:** 10.1371/journal.pgph.0002390

**Published:** 2023-09-22

**Authors:** Hafsa Lukwata, David Musoke, Solomon T. Wafula, John C. Ssempebwa

**Affiliations:** 1 Department of Epidemiology and Biostatistics, School of Public Health, College of Health Sciences, Makerere University, Kampala, Uganda; 2 Department of Disease Control and Environmental Health, School of Public Health, College of Health Sciences, Makerere University, Kampala, Uganda; Institute of Public Health Bengaluru, INDIA

## Abstract

Tobacco use is a leading cause of preventable deaths worldwide. Uganda enacted the Tobacco Control Act (TCA) 2015 to domesticate implementation of the World Health Organization (WHO) Framework Convention on Tobacco Control (FCTC) regulations. This study assessed the capacity to enforce the TCA and associated factors, as well as the level and barriers to its enforcement in Kampala, Uganda. A cross-sectional study using both quantitative and qualitative methods was employed. A structured questionnaire was used for quantitative data collection, and a WHO adapted capacity assessment open-ended guide was used for key informant interviews. Multivariable logistic regression was used to obtain odds ratios and 95% confidence intervals for the independent predictors for capacity to enforce the TCA. A total of 162 respondents from 5 institutions and six key informants were involved in the study. Findings established that only 23% (37/162) of the enforcers had the capacity to enforce the TCA. Male enforcers [OR = 0.16, 95% CI (0.05–0.55)], those who did not know when the law was enacted [OR = 0.19, 95% CI (0.07–0.52)], those with no plans to enforce the law [OR = 0.22, 95% CI (0.05–0.93)], and older enforcers (aged 31–40 years) [OR = 0.27, 95% CI (0.09–0.81)] were less likely to have the capacity to enforce the TCA. The level of enforcement of the TCA was mainly low to moderate for most of the institutions mandated to enforce it. Lack of knowledge about the law amongst the enforcers and general public, and inadequate funds were reported as major barriers to enforcement of the TCA. The capacity to enforce the TCA in Kampala was low. There is potential to enhance the capacity of enforcers through further dissemination of the Act, as well as sensitization of enforcers, institutional managers, and the general public about the legislation.

## Introduction

Tobacco use is a leading cause of preventable deaths globally. The use of tobacco is associated with an increased risk of cancer, cardiovascular and respiratory diseases, and unfavourable pregnancy outcomes [1–4]. Tobacco is the only legal substance that kills up to half of its consumers even when used as intended by its manufacturers [5]. According to the World Health Organization (WHO), at least 7 million people die each year from tobacco-related illnesses globally, with more than 6 million deaths due to direct tobacco use, and approximately 890,000 non-smokers dying due to exposure to second-hand smoke [6]. If current trends continue, tobacco related deaths are expected to increase to more than 8 million a year by 2030 [6]. Nearly 80% of the world’s one billion smokers live in low- and middle-income countries (LMICs) where 70% of the morbidity occur [7, 8]. In Uganda, tobacco use accounts for up to 25% of all deaths from non-communicable diseases (NCDs) [9]. The prevalence of smoking in adults is 16.7% in men and 3.4% in women [9], and the recent Global Youth Tobacco Survey indicates that 19.3% of young men and 15.8% of young women use tobacco [10]. According to statistics from the Uganda Cancer Institute, about 25% of lung cancer patients were tobacco users, and 16%, 13.7% and 12.6% of oral, stomach, and throat cancer patients respectively were former tobacco users [11].

In response to the global tobacco epidemic and related health consequences [12], the WHO Framework Convention on Tobacco Control (FCTC) was developed. This framework calls for comprehensive policies to address demand and supply reduction including legislation to eliminate tobacco smoking in all indoor public places and workplaces [13]. The WHO FCTC guidelines require member countries to designate and build the capacity of responsible authorities to spearhead the enforcement of compliance with these regulations [13]. To comply with these regulations, Uganda which ratified the WHO FCTC in 2007, enacted a comprehensive Tobacco Control Act (TCA) 2015 which came into effect in May 2016 [14]. This law prohibits smoking within 50 metres of all public places including workplaces, transport terminals, and other outdoor spaces. The legislation also bans tobacco advertising and promotions, as well as prohibits the supply, consumption and sale of tobacco products to and by minors below the age of 21 years. The TCA further bans the sale and use of flavoured tobacco products including shisha, as well as smokeless tobacco products such as Kuber and electronic nicotine delivery systems including e-cigarettes. According to the Act, owners of public places selling tobacco products are required to visibly display “No Smoking” signage at entries and in prominent positions within the premises [14]. The possible consequences for non-compliance to the TCA include penalty fines, imprisonment, or business suspension for six months [14]. These provisions of the Act should be enforced by authorised officers, who include Public Health Officers, National Environment Management Authority (NEMA) inspectors, police, customs officers, and any other individuals appointed by the Minister of Health [14].

Although tobacco control laws are enacted, there is no guarantee that they are enforced as required [15]. Evidence suggests that in addition to loopholes in legislation, one major barrier to the implementation of laws is the lack of capacity to implement them [16, 17]. Mismanagement and mishandling of laws can have adverse consequences on public health [15]. In Uganda, despite the enaction of the TCA, smoked and smokeless tobacco and their products are still widely supplied and openly sold in supermarkets, small retail shops, markets, kiosks, and roadside vendors [18]. In Kampala, the Kampala Capital City Authority (KCCA) has previously worked with the police and civil society to curb shisha users in various bars and restaurants in the city and surrounding suburbs. It has been shown that enforcers such as some police officers are not willing to participate in tobacco control activities citing a lack of adequate knowledge about the law [19]. The wide range of provisions in implementation of tobacco control, therefore, requires enforcers to be highly trained with comprehensive skills in dealing with the community. These skills include: the identification of tobacco control violations; analysis of reports; ability to testify in court; and the use of the enforcement information management system. To ensure enforcement of tobacco control laws, there is need for strong infrastructure, as well as sufficient human, financial and logistical resources [20, 21]. This study therefore assessed the capacity to enforce the TCA and associated factors, as well as the level and barriers to its enforcement in Kampala, Uganda.

## Methods

### Study design, setting and population

The study was cross sectional in design and employed a convergent mixed methods approach. The quantitative component involved a structured questionnaire while qualitatively, key informant interviews were held using an open-ended interview guide. The study was conducted in Kampala, the capital city of Uganda which has five divisions namely: Central, Nakawa, Kawempe, Rubaga, and Makindye. According to the 2014 census, Kampala had an estimated resident population of 1,507,080 people, with an annual growth rate of 2.02% [22]. However, Kampala has a daytime population of over 2.5 million people coming from mainly the surrounding Wakiso and Mukono districts, but also from other parts of the country. Kampala is located in the central region but has a very diverse ethnic population, with people from all over the country including refugees. Kampala is also home to several government agencies such as the Uganda National Bureau of Standards (UNBS), Uganda Revenue Authority (URA), and National Environment Management Authority (NEMA) that have the responsibility to implement the tobacco control law. Many non-governmental organisations (NGOs) working on tobacco control also have their head offices in Kampala, and there are many tobacco traders located in the city. The study population included authorized officers from different agencies as per the TCA 2015 who responded to the questionnaire including KCCA, police, UNBS, URA and NEMA. The respondents for interviews were key tobacco control personnel from these five institutions.

### Sample size and sampling

The sample size was calculated using Krejcie and Morgan (1970) sample size tables [23]. From the total population of enforcers in Kampala estimated at 339, 181 were selected for involvement in the study (Table 1).

**Table 1:** Sample size per institution.

Organisation	Number of enforcers	Sample Size
KCCA	178	95
NEMA (Environmental Police)	56	30
UNBS (Standards Inspectors)	45	24
URA (Customs enforcement officers)	15	8
Police (Criminal Investigation Department)	45	24
**Total**	**339**	**181**

Respondents from UNBS, URA and NEMA were randomly selected from their respective head offices depending on their availability on the days of data collection. KCCA enforcers were also selected randomly but ensuring an equal number were selected from each of the 5 divisions in Kampala. For the police, the major police station in each division was selected, and 5 respondents were randomly selected. The police respondents had to be from the anti-narcotics unit of the Criminal Investigation Division (CID) which is directly responsible for drug abuse related cases under which tobacco falls. For the qualitative interviews, a total of 6 key informants were interviewed based on institutions involved in the study. These key informants were purposively selected and included one from each institution such as the head of the enforcement unit, or the tobacco control focal person.

### Data collection and analysis

The questionnaire, which was administered by four Research Assistants, collected information on sociodemographic characteristics, knowledge and attitudes on the TCA, level of enforcement, perceived efficacy, and capacity to enforce. This questionnaire also captured the skills and experiences in enforcement including inspections, investigations, and operations, as well as on barriers related to implementation of the TCA. KoBoCollect v1.14.0a software was used to collect the questionnaire data on smart phones and handheld electronic tablets. To obtain expert information about the extent to which tobacco control law enforcement was done in Kampala, key informant interviews using an open-ended interview guide were conducted. The guide used was adopted from the WHO “five Ps” matrix, and had elements that addressed the current status of tobacco law enforcement, as well as highlighted the barriers to enforcement of the law. The themes of the guide included policy status, people (human resources) available / required, programme management including leadership and organisational structures, funds availability for tobacco control, and partnerships or inter-linkages with other organisations. The interviews were conducted at the respective organizations of the key informants in Kampala. Only the participants were present in the places where the interviews were conducted to ensure privacy. The interviews, which were audio recorded, transcribed, and thematically analysed, assessed the extent to which institutions were enforcing the law. Coding was done by the main research researcher (HL) to develop a code book. During analysis, related codes were grouped together to form sub-themes, and related themes grouped to form themes. Note taking was also done by the Research Assistants during the interviews. All data was collected in English, and a Research Supervisor supported the data collection process. The capacity to enforce the TCA was assessed using multiple factors related to individual knowledge, attitudes, motivation, self-efficacy, institutional support, and relationships with other related institutions. Other studies have found that multi-factor approach for measuring capacity has utility since each factor may look at a different dimension [24].

For the quantitative data, the outcome variable was capacity to enforce the TCA which was determined using four variables: 1. knowledge about the tobacco control law (5 questions were asked and responding appropriately to at least 3 of them was considered high knowledge, or low knowledge for 2 or less correct answers); 2. motivation to implement the law (binary: Yes / No); 3. having the right attitude (3 questions were asked and appropriate response to all of them was considered having the right attitude); and 4. having skills (2 questions asked—having been trained and having the confidence to implement: correct responses to both questions was considered appropriate). A respondent was considered to have capacity to enforce the Act if they scored as appropriately for the four variables as each of them had a contribution to the ability of the individual. To assess the level of enforcement of the different provisions of the TCA within an organisation, the respondents were asked how frequently they had participated in any tobacco control activity in the 6 months prior to the study. Mentioning 5 or more times was considered frequent; more than once but less than 5 times was considered occasional; once was considered rare; and none was considered never. All data was exported to Stata 13.0 (Statacorp Texas; USA) for cleaning and analysis. Descriptive statistics such as frequencies and proportions were performed for demographic characteristics of the respondents. Fisher’s tests were run to assess the association between sociodemographic characteristics and other variables with the capacity to enforce the law. Variables with p values less than 0.2 in the bivariable models were entered into a multivariable logistic regression to determine the independent predictors of the capacity to enforce the TCA. All significant variables were added into the model and the backward elimination method was used to eliminate variables starting with those with the highest p values until only significant variables were retained. The adjusted odds ratios and their corresponding 95% confidence intervals and p-values have been presented.

For qualitative data, the key informants were taken through a WHO adapted capacity assessment questionnaire guide. Factors affecting enforcement of the Act included: having a specific tobacco control policy; availability of a focal person and reports for the activities undertaken; frequency of execution of tobacco control activities; availability of funds and people (human resources); partnerships while undertaking tobacco control activities; and barriers to enforcement. Level of enforcement was considered: low when the institution had performed one or no tobacco related activity; moderate if only occasionally had activities; and high if there were frequent activities undertaken in the six months prior to the study. Responses were tabulated and compared across the institutions. Key statements that characterized major themes relating to study objectives were identified. During analysis of the qualitative data, an inductive approach was taken that enabled themes to be generated from the data. Since quantitative and qualitative methods asked different but related questions, the data from the two methods were treated as complementary. Results are presented together where appropriate for triangulation and to enhance confidence in the findings, as well as to contextualize the results.

### Ethical considerations

Ethical approval to conduct the study was obtained from the Makerere University School of Public Health Higher Degrees, Research and Ethics Committee. Permission was also sought from the tobacco control focal points at the target institutions prior to the study. Written informed consent was obtained from all respondents before their participation. Confidentiality and privacy were maintained throughout the study, and unique identification codes on the questionnaires and guides were used instead of names.

## Results

### Socio-demographic characteristics of respondents

A total of 162 enforcers (mean age 38 ± 10.8 years) participated in the study, representing a 90% response rate. There were more male health enforcers, with only 51 (31.5%) being females. More than half of the respondents had attained tertiary education 93 (57.4%), and worked for a period between 1–5 years 96 (59.2%). Among the respondents, 26 (15.5%) had ever used a tobacco product, and 4 (2.5%) were current users (Table 2).

**Table 2:** Respondents’ socio-demographic characteristics.

Variable	Number of participants (N = 162)	Percentage (%)
**Gender**		
Female	51	31.5
Male	111	68.5
**Age (years) (mean ± SD)**	38 ± 10.8	
21–30	53	32.7
31–40	90	55.6
41–50	12	7.4
51–60	17	4.3
**Length of time as enforcer (years)**		
1–5	96	59.2
6–10	45	27.8
11–15	10	6.2
≥ 16	11	6.8
**Highest education level**		
Secondary (ordinary) level	7	4.3
Secondary (advanced) level	62	38.3
Tertiary / University	93	57.4
**Tobacco use status**		
Never used	136	84.0
Ever used*	26	16.0
**Current user of tobacco**		
Yes	4	2.5
No	158	97.5

*Ever smoked includes current smokers

### Knowledge and perceptions of enforcers about the TCA

A total of 133 (82%) respondents had heard or knew about the TCA, and only 44 (27%) knew when it was passed. None of the respondents had got specific training on tobacco control enforcement, although 119 (74%) had received some form of training in law enforcement which varied in duration ranging from 2 days to 2 months. Most respondents 120 (95%) correctly mentioned at least one provision of the Act, and many knew the provision that prohibited smoking in public places. The majority of respondents 143 (89%) knew their institutions were responsible for the enforcement of the law, and 125 (78%) felt they had the motivation to enforce it. Less than half of the respondents 68 (43%) indicated that they had what it takes and were confident to enforce the TCA (Table 3). Overall, only 37 (23%) of the respondents had capacity to enforce the TCA in Kampala (Table 4).

**Table 3:** Respondents’ knowledge, perceptions and motivation on TCA enforcement.

Variable	Number of participants (N = 162)	Percentage (%)
**Knew / had heard about the law**		
Yes	133	82
No	29	18
**Knew when the TCA was passed**		
Yes	44	27
No	118	73
**Knew that they were responsible to enforce the TCA**		
Yes	143	89
No	19	11
**Knew correctly at least one provision of the TCA**		
Yes	120	75
No	42	25
**Had been trained as an enforcer**		
Yes	119	74
No	43	26
**Felt motivated to enforce**		
Yes	125	78
No	37	22
**Felt confident to enforce**		
Yes	68	43
No	94	57

**Table 4:** Composite score for capacity to enforce.

Variable	Scale	Percentage (%)
Knowledgeable about the TCA	3/5	27
Motivation towards the TCA	1	78
Attitudes towards the TCA	3/3	23
Having skills as enforcer	2/2	30
**Composite score for capacity to enforce**		**23**

### Capacity to enforce the TCA and associated factors

Fishers-exact tests found statistically significant differences between capacity to enforce the TCA with the following variables: knowledge about when the law was enacted; having or not having plans for enforcement; knowledge of whether the law had public support; and committed managers. More males 32 (86.5%) than females 5 (13.5%) had the capacity to enforce the TCA (p <0.01). More individuals who knew that their institutions had plans for tobacco enforcement (69%) had capacity to enforce the TCA as compared to those who did not know (p <0.01). Of the 37 respondents who had capacity to enforce the TCA, the majority 31 (83.8%) felt that the public was in support of the legislation. The majority of respondents who felt that their managers were committed to tobacco control (89.2%) had the capacity to enforce the TCA compared to 11% who felt that their managers were not (Table 5).

**Table 5:** Factors associated with capacity to enforce the TCA.

Characteristic	Overall [N, %]	Capacity to enforce		p-value
		No, n (%)	Yes, n (%)	
**Total**	162 (100)	125 (77.2)	37 (22.8)	
**Gender**				
Male	111 (68.5)	79 (63.2)	32 (86.5)	0.01*
Female	51 (31.5)	46 (36.8)	5 (13.5)	
**Age (years)**				
21–30	53 (32.7)	39 (31.2)	14 (37.8)	0.31
31–40	90 (55.6)	71 (56.8)	19 (51.4)	
41–50	12 (7.4)	11 (8.8)	1 (2.7)	
51–60	7 (4.3)	4 (3.2)	3 (8.1)	
**Education level**				
Secondary (ordinary) level	7 (4.3)	5 (4.0)	2 (5.4)	
Secondary (advanced) level	62 (38.3)	49 (39.2)	13 (35.1)	0.80
Tertiary / University	93 (57.4)	71 (56.8)	22 (59.5)	
**Years worked as an enforcer**				
1–5	96 (59.3)	73 (58.4)	23 (62.2)	0.36
6–10	45 (27.8)	34 (27.2)	11 (29.7)	
11–15	10 (6.2)	10 (8.0)	0 (0.0)	
≥ 16	11 (6.8)	8 (6.4)	3 (8.1)	
**Ever used tobacco**				
No	136 (84.0)	107 (86.3)	29 (78.4)	0.24
Yes	26 (16.0)	17 (13.7)	9 (21.6)	
**Knew when the TCA was passed /enacted**				
Yes	44 (27.2)	25 (20.0)	19 (51.4)	<0.01*
No	118 (72.8)	100 (80.0)	18 (48.6)	
**Law will achieve its objective**				
Agreed	124 (77.5)	91 (74.0)	33 (89.2)	0.07
Disagreed	36 (22.5)	32 (26.0)	4 (10.8)	
**Had responsibility to implement the law**				
Not sure	7 (4.4)	7 (5.7)	0 (0.0)	0.23
No	10 (6.3)	9 (7.3)	1 (2.7)	
Yes	143 (89.4)	107 (87.0)	36 (97.3)	
**Plans for enforcement**				
Yes	119 (74.4)	85 (69.1)	34 (91.9)	<0.01*
No	41 (25.6)	38 (30.9)	3 (8.1)	
**Public support for the TCA**				
No	50 (31.2)	44 (35.8)	6 (16.2)	0.02*
Yes	110 (68.8)	79 (64.2)	31 (83.8)	
**Commitment of managers towards tobacco control**				
No	39 (24.1)	35 (28.0)	4 (10.8)	0.047*
Yes	123 (75.9)	90 (72.0)	33 (89.2)	

* Statistically significant

### Predictors of capacity to enforce the TCA

Multiple regression analysis found that respondents who had no plans of enforcement were 78% less likely to have capacity to enforce the TCA as compared to those who agreed to having plans [OR = 0.22, 95% CI (0.05–0.93, p = 0.04)]. Respondents who did not know when the law was passed were 81% less likely to have capacity to enforce the TCA compared to those who knew [OR = 0.19, 95% CI (0.07–0.52, p < 0.01)]. Compared to those aged 21–30 years, respondents aged 31–40 years were 73% less likely to have capacity to enforce the TCA [OR = 0.27, 95% CI (0.09–0.81, p = 0.02)]. The significant association between perception of public support for the TCA and capacity to enforce it was lost after adjustment for covariates (Table 6).

**Table 6:** Predictors of capacity to enforce TCA.

Predictor variables	Unadjusted OR (95% CI)	p-value	Adjusted OR (95% CI)	p value
**Gender**				
Male	1		1	
Female	0.27 (0.10–0.74)	0.01	0.16 (0.05–0.55)	<0.01*
**When was the TCA passed**				
Knew	1		1	
Did not know	0.24 (0.11–0.53)	<0.01	0.19 (0.07–0.52)	<0.01*
**The law will achieve its objective**				
Agreed	1		1	
Disagreed	0.34 (0.11–1.05)	0.06	0.59 (0.17–2.10)	0.42
**Had plans for enforcement**				
Yes	1		1	
No	0.20 (0.06–0.68)	0.01	0.22 (0.05–0.93)	0.04*
**Public support for the TCA**				
No	1		1	
Yes	2.88 (1.11–7.43)	0.03	2.48 (0.84–7.33)	0.10
**Commitment of managers on tobacco use control**				
No	1		1	
Yes	3.15 (1.04–9.57)	0.04	0.86 (0.23–3.17)	0.82
**Age (years)**				
21–30	1		1	
31–40	0.76 (0.34–1.67)	0.49	0.27 (0.09–0.81)	0.02*
41–50	0.28 (0.03–2.38)	0.24	0.09 (0.01–1.11)	0.06
51–60	2.09 (0.41–10.52)	0.37	2.39 (0.27–20.75)	0.43
**Ever used tobacco**				
No	1		1	
Yes	1.84 (0.72–4.74)	0.20	1.24 (0.34–4.55)	0.74

* Statistically significant at multivariate analysis

### Enablers and barriers to enforcement of the TCA

Respondents who felt it would be easy to enforce the TCA cited high institutional authority to implement any law 30 (56%) and high public support 17 (31.5%) as enablers. Enforcers who expected enforcement of the TCA to be difficult cited barriers such as lack of knowledge about the significance of the law and the dangers of tobacco 30 (27%), as well as political interference and lack of political will 29 (26%) (Table 7).

**Table 7:** Perceived enablers and barriers to enforcement of the TCA.

Response	Number of participants (N = 162)	Percentage (%)
Perceived enablers* **(n = 50)**		
Institutional authority	30	55.6
Public support	17	31.5
Tobacco being dangerous to health	4	7.4
Not so many users of tobacco	3	5.6
Perceived barriers to enforcement of TCA* **(n = 97)**		
Lack of knowledge by enforcers and the public	30	27.0
Political interference and lack of political will	29	26.1
Lack of government support	22	19.8
Corruption	14	12.6
Tobacco being a livelihood for some people	7	6.3
Lack of coordination between government departments	9	8.1

* Multiple responses were accepted

The major barriers to implementation of the TCA included: lack of adequate funds to manage tobacco programme activities (72%), lack of information and awareness about the law and on dangers of tobacco use among the enforcers and general population (66%), lack of institutional support / prioritisation (50%), corruption (17%), and poor infrastructure (7%).

The qualitative findings revealed lack of prioritization of the enforcement sections of the institutions where enforcers were too few in relation to their mandate hence found challenges in the community. In addition, institutional infrastructure was inadequate to implement the TCA especially with NEMA which lacked the structures at local government level.

*“There is shortage in manpower and we have had episodes of lawlessness and violence from the community*. *It is possible to be overpowered by community members even if you move with the police”*.

Key Informant, KCCA

Corruption was also highlighted as a major barrier to enforcement of the TCA. This included enforcers receiving bribes, failure to impound or arrest offenders, and interference by politicians and business owners including tobacco manufacturers.

*‘There is a lot of political interference as well as from the tobacco industry in the implementation of the Tobacco Control Act in Kampala*. *Unless these challenges are addressed*, *it will be hard to adequately implement this law*.*’*

Key Informant, KCCA

### Level of enforcement of provisions of the TCA

Enforcers were asked whether they had participated in tobacco control activities, and how often they did so in the 6 months prior to the study. Less than half of the respondents 72 (44%) reported at least once (rarely), 38 (23%) reported five and more times (frequently), while 52 (33%) had never participated in any tobacco control activity. From the key informant interviews, 4 of the 6 institutions were implementing a specific tobacco control policy or provision, while 5 had the required human resources for tobacco control activities. However, only 3 institutions had trained some of their officers on tobacco control. Only KCCA and MOH had dedicated tobacco control focal persons. In addition, only KCCA had dedicated funds for tobacco control from a five-year ongoing project on promoting smoke free cities. Five of the institutions required to enforce the TCA were implementing some tobacco control activities. However, these institutions had only a few of the required elements in place to enable them to fully enforce the law in Kampala. The major thematic areas identified in the conceptual framework to assess institutional capacity to enforce the TCA, and the extent to which the different organisations had enforced it is summarised in Table 8.

**Table 8:** Status of different organisations on implementation of tobacco control.

	Police	KCCA	NEMA	UNBS	URA	MOH
**Implementation of a specific policy or provision**	None	Smoke free in public places	None	Developing and monitoring of standards for cigarettes	Monitoring illicit trade of tobacco products	Coordination of implementation of tobacco control interventions
**Human resources (HR) issues**	Human resource available but not trained in tobacco control	Available but not trained in tobacco control	Not available	Trained human resource but not enough enforcers	Not well-trained human resource, and not enough enforcers on tobacco control	Well trained but very few to manage the mandate
**Programme management**	No focal person, occasionally engage in tobacco control activities	Project management unit available, have planned daily activities	No focal person No activities on tobacco control	No focal person Occasionally engage in tobacco control activities	No focal person. Have planned activities	There is a focal person. Have planned activities but not on a daily basis
**Availability of funds**	No dedicated funds	Having funds from international agencies for five years	No dedicated funds	No dedicated funds	No dedicated funds	No dedicated funds but there are funders for specific activities
**Availability of partnerships**	Partners with civil society organisations and other government departments such as KCCA	Works with mainly police but also government departments such as MOH	Works with public health inspectors	Works with police	Works with mainly police and Uganda People Defense Forces (UPDF)	Works closely with NGOs and other government departments

## Discussion

The study assessed the capacity of enforcers and level of enforcement of the Tobacco Control Act 2015 in Kampala, Uganda. Our findings show that most enforcers had heard about the TCA received some training, trained as enforcers, and felt motivated to enforce the legislation. However, less than a quarter of the enforcers had capacity to enforce the law. Enforcers who were female, older (31–40 years), did not know when the law was enacted, and those who had no plans to enforce the law were less likely to have capacity to enforce the TCA 2015. The study findings show that despite enactment of the TCA in 2015, more effort is needed to ensure that enforcers have the capacity to enforce the legislation for better public health outcomes in Kampala.

In our study, only 23% of the enforcers had capacity to enforce the TCA which is of a great concern to public health. This low capacity is likely to affect the ease and appropriateness with which the TCA is implemented in Kampala and other parts of the country. This finding highlights the need to ensure enforcers receive specific training about the TCA, and to be provided with sufficient support to enhance the law being implemented. One of the key aspects affecting capacity to enforce laws is attitude. This is a tendency to react positively or negatively towards a certain issue or idea or person, and one’s attitude influences their choice of action, as well as responses to challenges, incentives, and rewards [25]. From our results, less than 1 in 4 respondents had positive attitudes towards the TCA, which can affect how these enforcers implement the law. Our study also indicated that most respondents were motivated to enforce the law. Motivation results from the interaction of both conscious and unconscious factors such as the intensity of desire or need, incentive or reward, value of the goal, and expectations of the individual and of their peers affecting behaviour [26]. Workers who are self-motivated do not need or be lured by incentives or rewards since they are confident in their abilities, and personally identify with their role within the organisation.

Nearly three quarters of the enforcers had received some form of training regarding their work. In our study, training was not a significant predictor of capacity implying that may not necessarily translate into an equally high rate of capacity to implement the law. This finding is in agreement with other researchers who found that although implementer training is essential as it improves the knowledge and attitudes, training alone is not sufficient to ensure implementation [17]. In addition to training, other factors that are likely to increase the capacity of enforcers to implement the law include availability of finances and other logistics such as transportation, as well as institutional support. The WHO FCTC guidelines propose that member states should specifically have tobacco control enforcement officers adequately trained in the content of the law, enforcement procedures, and methods for interacting with violators and the public so as to promote acceptance of the law among the regulated community [13]. Unfortunately, none of the enforcers had received specific tobacco control trainings. This finding justifies the need to train enforcers in tobacco control to enhance enforcement of the TCA.

Findings from our study indicate that females were less likely to have capacity to enforce the TCA than male enforcers. Some studies have indicated that more male enforcers were preferred because of the need for them to work in awkward hours of the night for enforcement of smoke free places in the hospitality sector including bars and restaurants [27]. Male enforcers may therefore have more capacity to bring to order smokers who tend to go out in the open space as it gets colder in the later hours of the night thus the tendency to abrogate the rules by smoking indoors [20]. Nevertheless, with more females carrying out more male-dominated roles in society [28, 29], their capacity to also enforce the TCA should be supported. For example, law enforcement teams implementing the law at night could include security personnel such as the police to make it more favourable for females to take part. Therefore, initiatives to enhance to the capacity of enforcers of the TCA should consider both males and females to support enforcement of the Act.

It was found in our study that compared to younger enforcers (aged 21–30), those aged 31–40 years were less likely to have the capacity to enforce the TCA. This is concurrent with findings among public health enforcers which found that younger people had higher enforcement capacity than their older counterparts [15]. The younger people are perhaps expected to be more energetic and still enthusiastic to work as opposed to the older ones who could have developed biases and prejudices over time. Whereas this study found that years worked as an enforcer was not a significant predictor of capacity to enforce the TCA, a Malaysian study revealed that public health officers who had worked for less than 4 years were competent but less effective as compared to the older ones [15]. However, being in service more than 9 years did not improve enforcement in that study. Fatigue due to routine work for those who have worked for a long time may also limit their capacity. It may be imperative to consider duty and station rotations of enforcers of legislation so as to improve their performance [30].

Enforcers who reported having plans for TCA enforcement were more likely to enforce the law than those who did not. This can perhaps be explained by the fact that once enforcement plans are in place, there is more likelihood for both human and financial resources to be availed to support execution. In addition to availability of plans for enforcement, supportive management can also facilitate the process. Having a supportive manager was marginally statistically significant regarding enforcement of the TCA in our study. In a study conducted in Texas, USA capacity to implement school based tobacco control measures was higher for those with supportive managers [17]. In addition, the Malaysia study on enforcement capacity of public health enforcers found that superior support was positively correlated with higher enforcement capacity [15]. It is therefore of paramount importance that managers of enforcement officers provide the necessary support to facilitate implementation of the TCA.

Regarding level of enforcement, all five institutions involved in the study by virtue of their mandate were supposed to implement at least one specific tobacco control policy or provision of the TCA. However, KCCA is mandated to implement all provisions of the law without any reservations. Police and NEMA did not have any planned tobacco control activities, although they believed they had a role to play in implementing the TCA. NEMA did not have any structures at the local government level and hence had no tobacco control activities in the previous six months. Instead, NEMA worked with other public officers such as for environmental protection in the districts but not tobacco control. The police were only called upon to offer protection during enforcement operations by other institutions such as KCCA. In addition, the police did not own any of those activities and thus did not have tobacco-specific reports. Therefore, the level of implementation of the TCA was low for these institutions. All institutions involved in the study apart from NEMA had some officers who could specifically enforce the TCA. However, the numbers and mix of the people required to enforce the law were inadequate. Lack of adequate human resources was reported as hampering the implementation of tobacco control activities by all institutions involved. This problem was also reported by other studies on tobacco control implementation which attributed it to lack of prioritisation by the government and institutional support [31]. To ensure adequate enforcement of the TCA, there is a need to ensure sufficient human resource is available in the various institutions involved.

All five institutions involved in the study reported having a degree of partnership with other government or non-government organisations. This was a positive practice as it is known that effective tobacco control is dependent on the balanced implementation of demand and supply reduction strategies through intersectoral collaboration involving various stakeholders including government departments, ministries and the private sector [32]. Partnerships in law enforcement may take a proactive approach where agencies actively plan for potential situations, share information, discuss potential issues, as well as establish joint protocols and lines of communication. Such collaborative approaches have been demonstrated in several studies to play a significant role in the enforcement of legislation in public health [28, 33, 34]. Intersectoral collaboration should therefore be emphasized as a strategy for ensuring that various stakeholders contribute to the enforcement of tobacco control legislation.

Inadequate funds were reported as a major reason impeding the implementation of the TCA in our study. Funds are crucial in the implementation of any legislation as they support various aspects including but not limited to recruitment and training of enforcers, increasing public awareness about the law, as well as logistics during enforcement including transportation. A similar challenge of funding was reported in a study carried out in California, USA about implementing smoke-free bans in bars and restaurants [28]. In addition, a study carried out in India mentioned lack of funds as an important barrier to effective enforcement of the law [31]. Another study showed that inadequate resources led many communities not to undertake systematic and/or proactive initiatives to enforce tobacco control laws [32]. It has been proposed that managers need to explore innovative avenues and integrate tobacco control with other health-related programmes at different levels to overcome the funding problem [26]. Government and other stakeholders need to ensure sustainable funding is available to support the implementation of the TCA in Kampala (and other parts of the country).

Lack of knowledge and awareness by the community about tobacco control laws and the dangers of tobacco use was a major barrier to enforcement of the TCA in our study. In a related study carried out in India, lack of awareness was highlighted as a significant problem in the enforcement of tobacco control legislation [31]. Consequently, the India study advocated for the utilisation of various communication channels such as television, media, advertisements for health warnings, and health education as part of the prevention strategy. Lack of knowledge on tobacco control and the need for skills to improve their enforcement has also been highlighted in other studies [31, 35]. Knowledge is one of the factors that affect a person’s perceptions, competencies, and willingness to commit to undertaking an activity [36]. Multifaceted educational strategies in conjunction with community-based campaigns can therefore bring substantial change in tobacco use and ease compliance with the law [37]. Creating public awareness and improving access to information regarding the adverse health, economic and environmental consequences of tobacco production and consumption can minimise tobacco use prevalence.

About half of the respondents mentioned that lack of support and prioritization of tobacco control by both technical and political leaders negatively affecting enforcement of the TCA. Studies in the USA have reported that politicians may fail the law right from the time of drafting to its implementation by undermining the strategies, or failing compliance [28, 33]. Politicians, in addition to being central in drafting legislation, are also mandated to mobilise communities for behavioural change. Funds and other resources can be made available, and pertinent issues can be brought to the agenda at different levels once the leaders are in support of a programme [27, 38]. Corruption was also noted as one of the barriers to enforcement of the TCA in our study and was in form of receiving bribes, and failure to impound or arrest an offender. A bribe will usually persuade authorities to deter enforcement action against wrongdoers and turn it towards their incentive. This creates a conflict of interest leading to poor or non-enforcement of laws and policies as has been demonstrated elsewhere [39, 40]. Therefore, full government support with no political interference is of paramount importance to support the implementation of tobacco control legislation such as the TCA.

Our study was limited by the fact that it was cross-sectional hence we could not make causal inferences. In addition, a relatively small number of individuals participated in the qualitative component of the study. It is also worth noting that the results may not be generalizable to other districts in the country because of the peculiar dynamics within Kampala. It is also worth noting that although institutional / programmatic support and readiness are important factors in enforcement of the TCA, they were not included in the composite score that measured enforcement capacity. Nevertheless, the results of the study provide evidence on issues surrounding enforcement of the TCA in Kampala which can inform other studies elsewhere in the country. Our study adds to the much-needed body of knowledge regarding the assessment of enforcers’ capacity as a vital step in informing the development and implementation of an enforcement strategy for tobacco control in the country. Since law enforcement is determined by a myriad of factors of which enforcers’ capacity is key, further research should be done to quantify the contribution of enforcers in reducing tobacco use among different segments of the community.

## Conclusion

The study found that most enforcers lacked specific knowledge about the TCA, and did not have the required skills to enforce it. The level of enforcement of the law was mainly low to moderate, thus a need to build institutional capacity including supportive managers to ensure effective implementation. The capacity to enforce the TCA was low, although the potential for improvement existed since most enforcers were motivated to enforce it. There is need to enhance the dissemination of the law, as well as ensure sensitisation of enforcers, institutional managers, and the general public about the harmful effects of tobacco use.

## Supporting information

S1 Dataset Study dataset. (XLSX)
